# Evaluation of Wearable Technology in Dementia: A Systematic Review and Meta-Analysis

**DOI:** 10.3389/fmed.2020.501104

**Published:** 2021-01-11

**Authors:** Alanna C. Cote, Riley J. Phelps, Nina Shaafi Kabiri, Jaspreet S. Bhangu, Kevin “Kip” Thomas

**Affiliations:** ^1^Department of Anatomy and Neurobiology, Boston University Medical Center, Boston, MA, United States; ^2^Department of Genetics and Genomic Sciences, Icahn School of Medicine at Mount Sinai, New York, NY, United States; ^3^Division of Geriatric Medicine, Department of Medicine, Western University, London, ON, Canada

**Keywords:** technology, geriatrics, cognition, sleep, wearable

## Abstract

**Background:** The objective of this analysis was to systematically review studies employing wearable technology in patients with dementia by quantifying differences in digitally captured physiological endpoints.

**Methods:** This systematic review and meta-analysis was based on web searches of Cochrane Database, PsycInfo, Pubmed, Embase, and IEEE between October 25–31st, 2017. Observational studies providing physiological data measured by wearable technology on participants with dementia with a mean age ≥50. Data were extracted according to PRISMA guidelines and methodological quality assessed independently using Downs and Black criteria. Standardized mean differences between cases and controls were estimated using random-effects models.

**Results:** Forty-eight studies from 18,456 screened abstracts (Dementia: *n* = 2,516, Control: *n* = 1,224) met inclusion criteria for the systematic review. Nineteen of these studies were included in one or multiple meta-analyses (Dementia: *n* = 617, Control: *n* = 406). Participants with dementia demonstrated lower levels of daily activity (standardized mean difference (SMD), −1.60; 95% CI, −2.66 to −0.55), decreased sleep efficiency (SMD, −0.52; 95% CI, −0.89 to −0.16), and greater intradaily circadian variability (SMD, 0.46; 95% CI, 0.27 to 0.65) than controls, among other measures. Statistical between-study heterogeneity was observed, possibly due to variation in testing duration, device type or patient setting.

**Conclusions and Relevance:** Digitally captured data using wearable devices revealed that adults with dementia were less active, demonstrated increased fragmentation of their sleep-wake cycle and a loss of typical diurnal variation in circadian rhythm as compared to controls.

## Introduction and Background

Dementia has been identified by the World Health Organization as a global priority for public health and social care in the twenty-first century (1). Advances in the molecular and genetic understanding of neurodegenerative disease has contributed to improved diagnostic paradigms and helped to foster a new era of personalized medicine for patients with dementia. This has coincided with the advancement in biological drug development for targeted therapies. These therapies have reflected the maturation in the scientific understanding of dementia that goes beyond raw measurement of cognitive performance. As a case in point a recent review of active clinical trials had shown that 14 biological treatments have targeted neuropsychiatric and behavioral symptoms as primary end-points. Challenges remain in capturing the heterogeneity of the clinical course experienced by individuals with dementia and translating these into meaningful end-points.

Technological advances using accelerometers, gyroscopes, and other motion detectors housed in mobile platforms may eventually present a cost-effective way to measure disease burden and deploy personalized treatments (2). Wearable devices that can continuously monitor physiological measures over extended periods, for example in the patient's home, provide unique information not attainable with traditional in-clinic monitoring and hold particular appeal in dementia populations (3). Advances in technology have made these devices increasingly affordable and user friendly but have been limited by methodological challenges. Specifically, their high resolution and sensitivity leaves them susceptible to noisy interference, complicated and time-consuming analytical techniques are required to derive clinically meaningful endpoints from the large amounts of data they produce, and the lack of standards has led to isolated “islands of expertise” (4).

The flexibility of wearable platforms has resulted in a variety of different uses including monitoring of gait, motion tracking, and sleep and circadian rhythm assessment (5). The ability to identify objective measurements of specific endpoints with respect to individual and group-wise subject performance, captured in real-time at various settings including at home, provides ecological validity that would otherwise be lost in laboratory settings. The main question that we had aimed to address was the potential for wearable devices to provide information on the behavioral and neuropsychiatric fluctuations inherent in the clinical course of dementia. The ability to accurately and objectively measure these fluctuations can provide researchers with viable digital surrogate end-points for use in clinical trials. We undertook a systematic review and meta-analysis to evaluate the utility of wearable technology in patients with dementia for the measurement of these neurophysiological parameters. The objective of this analysis was to systematically review studies employing wearable technology in patients with dementia by quantifying differences in digitally captured neurophysiological endpoints.

## Literature Selection Criteria

### Data Sources and Search Strategy

Five electronic databases were searched including Cochrane, EMBASE, PubMed, PsycInfo, and IEEE. Searches were performed for Cochrane, PsycInfo, and IEEE on October 31, 2017. A PubMed search was performed on October 25, 2017, and an Embase search on October 27, 2017. A combination of Medical Subject Headings and search terms were constructed by the authors (RP, AC, and JB) in collaboration with a librarian. The Search Terms provides an outline of the search strategy for PubMed only.

### Search Terms. Systematic Review Search Strategy: PubMed

1. “Dementia”[Mesh]
2. dementia
3. (frontotemporal dementia)
4. (vascular dementia)
5. (Alzheimer^*^ disease)
6. (Parkinson^*^ disease)
7. (lewy body)
8. Creutzfeldt-Jakob
9. 1 or 2 or 3 or 4 or 5 or 6 or 7 or 8
10. “Technology”[Mesh]
11. (wearable device)
12. (assistive technology)
13. (wearable technology)
14. on-body
15. bracelet
16. GPS
17. actigraphy
18. accelerometer
19. (galvanic skin response)
20. biosensor
21. sensor
22. gyroscope
23. watch
24. necklace
25. harness
26. strap
27. patch
28. camera
29. chip
30. (step counter)
31. pouch
32. armband
33. node
34. 10 or 11 or 12 or 13 or 14 or 15 or 16 or 17 or 18 or 19 or 20 or 21 or 22 or 23 or 24 or 25 or 26 or 27 or 28 or 29 or 30 or 31 or 32 or 33
35. 9 and 3.

### Types of Studies

We included observational studies reporting primary data in a peer-reviewed scientific journal. Studies had to include participants with a mean age ≥50 years and did not include any direct intervention (i.e., drug, vitamin, supplement, exercise, cognitive, or behavioral intervention). Studies published before 1970 or translated to English were excluded. Studies that did not provide descriptive statistics for a physiological outcome were excluded. Conference abstracts, review papers, case reports, letters, opinion pieces, editorials, article comments, or corrections were excluded.

### Type of Exposure

We included all-cause dementia (any dementia subtype) as our exposure (6). Exact search terms for dementia subtypes included can be found in the search strategy (Search Terms). As we included studies from 1970 onwards, diagnostic criteria for the diagnosis of dementia differed between studies and is summarized for each study in Table 1.

**Table 1:** Patient setting and diagnostic criteria for 48 observational studies testing wearable technology in participants with dementia.

**Source**	**Diagnostic criteria**	**Setting**
Aharon-Peretz et al. (7)	DSM III-R (8)/NINCDS-ADRDA (9)	Not stated
Ahmed et al. (10)	McKhann et al. (11)/Gorno-Tempini et al. (12)/Rascovsky et al. (13)	Community (home)/In Lab
Anderson et al. (14)	Neary criteria (15)	Community (home)
Brown et al. (16)	Medical record diagnosis	Nursing home
Carvalho-Bos et al. (17)	NINCDS-ADRDA (9)/DSM-IV (18)	Nursing home
David et al. (19)	DSM-IV (18)	Out-patient clinic
David et al. (20)	NINCDS-ADRDA (9)	Out-patient clinic
Eggermont and Scherder (21)	Medical record diagnosis	Nursing home
Fetveit and Bjorvatn (22)	Clinical Dementia Rating (CDR) scale (23)	Nursing home
Fleiner et al. (24)	ICD-10 (25)	Psychiatric hospital
Gehrman et al. (26)	Medical record diagnosis/NINCDS-ARDA (9)	Nursing home
Ghali et al. (27)	DSM-III-R (8)	Dementia treatment Evaluation facility
Harper et al. (28)	NINCDS-ADRDA (9)	Hospital/clinical research center
Harper et al. (29)	NINCDS-ADRDA (9)	Hospital/clinical research center
Hatfield et al. (30)	DSM-IV (18)/ NINCDS-ADRDA (9)	Community (home)
Hooghiemstra et al. (31)	DSM-V (32)/NINCDS-ADRDA (9)/Neary Criteria (15)/McKeith et al. (33)/Pohjasvaara et al. (34)	Not stated
Ijmker and Lamoth (35)	Clinician/ medical record diagnosis	In laboratory
Iwata et al. (36)	DSM-IV (18)/NINCDS-ARDA (9)	Not stated
James et al. (37)	NINCDS-ARDA (9)	Community (home)
Kodama et al. (38)	DSM-III-R (8)	Community (home)
König et al. (39)	International working group−2 criteria (IWG-2) (40)	Memory clinic
Kuhlmei et al. (41)	NINCDS-ADRDA (9)/NINDS-AIREN (42)	Not stated
La Morgia et al. (43)	NINCDS-ADRDA (9)	Not stated
Lamoth et al. (44)	Alzheimer's association criteria	Clinic/hospital
Landolt et al. (45)	Autopsy or biopsy confirmation	Hospital/ nursing home
Lee et al. (46)	NINCDS-ADRDA (9)	Not stated
Leger et al. (47)	DSM-V (32)/ NINCDS-ADRDA (9)/MMSE ≤ 25 and ≥15 (48)/CDR (Score of 0.5, 1, or 2) (23)	Out-patient clinic
McCurry et al. (49)	Family physician/medical record diagnosis	Community (home)
Merrilees et al. (50)	Neary criteria for frontotemporal lobar degeneration (51)	Community (home)
Most et al. (52)	NINCDS- ADRDA (9)	Not stated
Moyle et al. (53)	Medical record diagnosis	Long-term care facility
Mulin et al. (54)	NINCDS- ADRDA (9)	Community (home)
Murphy et al. (55)	MMSE < 23 (48)	Nursing home
Olsen et al. (56)	Medical record diagnosis or MMSE < 25 (48)	Nursing home/community (home)
Paavilainen et al. (57)	CDR > 0.5 (23)/ MMSE < 20 (48)	Nursing home
Pollak and Stokes (58)	Mattis dementia rating scale total score < 123 (59) Mattis dementia rating scale memory score < 19 (59)	Community (home)
Rindlisbacher and Hopkins (60)	DSM-III-R (8)	Hospital
Schwenk et al. (61)	NINCDS-ADRDA (9)/NINDS-AIREN (42)	Community (home)
Valembois et al. (62)	DSM-IV (18)	Hospital
van Alphen et al. (63)	Medical record diagnosis	Community (home)/nursing home
van Someren et al. (64)	DSM-III-R (8)/NINCDS-ADRDA (9)	Community (home)/nursing home
Varma and Watts (65)	NINCDS-ADRDA (9)	Community (home)
Viegas et al. (66)	DSM-IV (18)/MMSE ≤ 24 (48)	Nursing home
Volicer et al. (67)	NINCDS-ADRDA (9)/DSM-III-R (8)	Hospital
Wams et al. (68)	NINCDS-ADRDA (9)	Community (home)
Weissova et al. (69)	NINCDS-ADRDA (9)	Community (home)
Wirz-Justice et al. (70)	DSM –IV (18)	Hospital
Yesavage et al. (71)	NINCDS-ADRDA (9)	Community (home)

### Types of Outcome Measures

We included studies which provided physiological data as measured by wearable technology. Wearable technology was defined as a non-implantable, body-fixed sensor technology designed to monitor for >24 h and to not interfere with the wearer's normal activity (5, 72). By this definition, studies using finger-based pulse oximeters, blood pressure monitors, galvanic skin response sensors, functional near-infrared spectroscopy (fNIRS), and electroencephalograms (EEG) were excluded. Where studies included measurement devices other than a wearable device, only data from the wearable device was included in the final analysis.

### Methods for Literature Secondary Screening

#### First Selection: Abstract Screening

**Figure 1:**
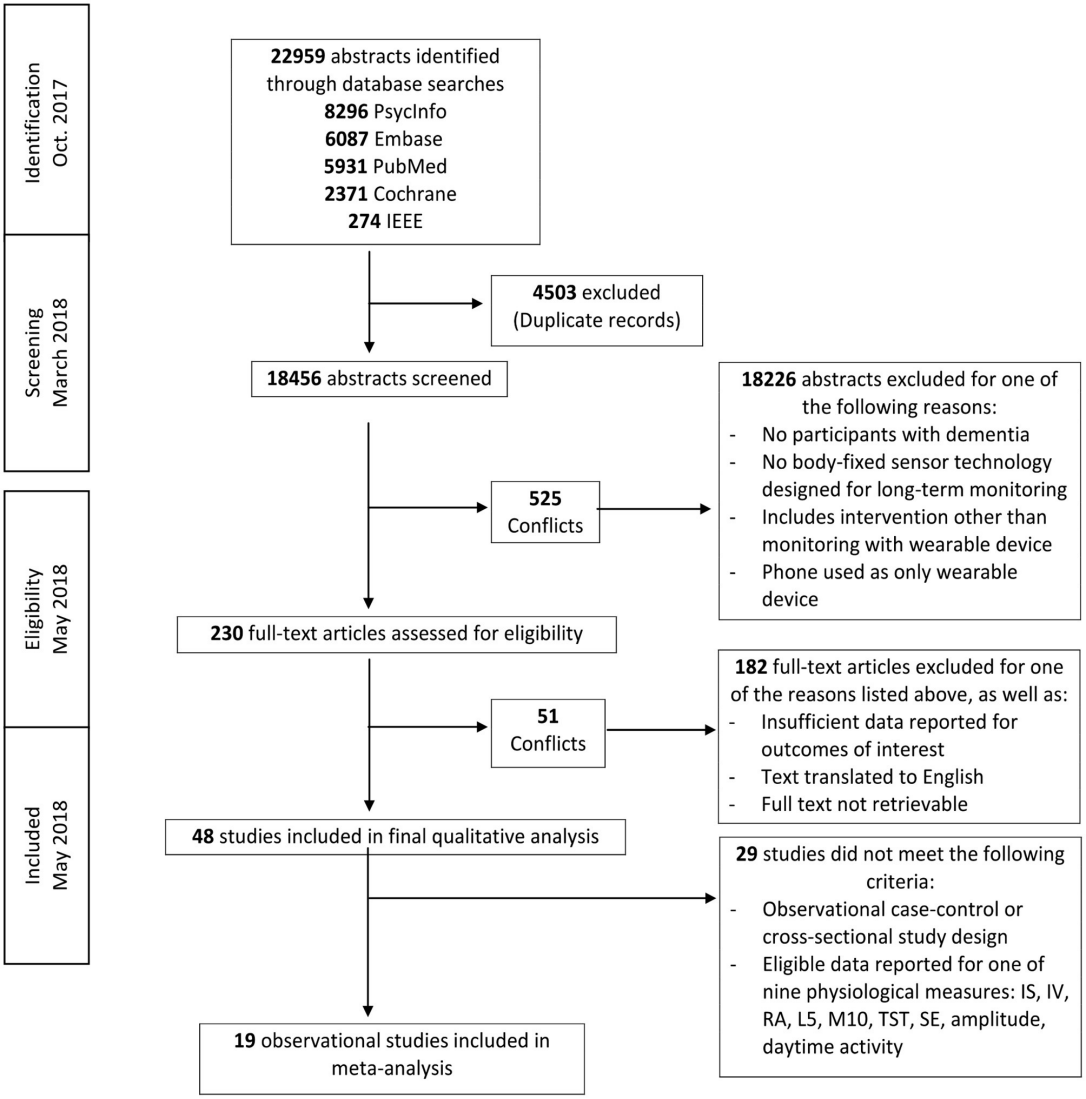
Literature search flow diagram.

Two authors (RP and AC) independently screened each record by title and abstract according to eligibility criteria. Eighteen thousand four hundred fifty-six abstracts were included in the initial screening process. There were 525 disagreements in abstract selection between the two reviewers. Conflicts were resolved by two additional authors (NS and JB) using the inclusion and exclusion criteria and definitions outlined in Figure 1.

#### Final Selection

Two hundred thirty articles were eligible for full text review. Two authors (RP and AC) independently determined eligibility of each article for inclusion. In cases of disagreement or conflict, senior authors (NS, JB, and KT) determined whether the study met eligibility criteria. Forty-eight articles were included in the final systematic review.

### Data Collection

Data was extracted by three authors (JB, RP, and AC). Information extracted from each publication is provided in **Table 6**. To assess the methodological quality of included studies, we used the checklist provided by Downs and Black (73). A total quality score is provided for each study in **Table 6** (maximum score = 32).

### Statistical Analyses

Data analysis was performed using Stata/SE (StataCorp LP, Texas, Version 15). The age and number of included participants per study, as well as general study results are provided in **Table 6**. Initial synthesis of qualitative data revealed a number of common endpoints reported consistently by authors. Subsequent meta-analyses included only observational case-control or cross-sectional studies that presented data for these commonly reported endpoints (**Table 7**).

Meta-analyses were conducted using the standardized mean difference (Hedges' g). Hedges' g values ≤0.20, >0.20 but <0.80, and ≥0.80 were considered small, moderate, or large, respectively between controls and participants with dementia (74). For each single or combined effect size, a positive value indicated a higher mean value of that variable in participants with dementia than in healthy volunteers. Some publications contained two subgroups of dementia participants. A fixed effects meta-analysis was performed on the dementia subgroups within each of these studies to compute a composite effect size and variance (75). This composite effect was used in the across-study random effects analysis.

Across-study heterogeneity was investigated using the Cochran's *Q*-test and *I*^2^ statistic. Cochran's Q test was performed using the weighted method of moments method (75). Cochran's Q statistic was considered significant at *p* < 0.10. *I*^2^-values of 25, 50, and 75% were considered indicative of low, moderate, and high heterogeneity, respectively (76).

For each analysis, a funnel plot of standardized mean differences was constructed, and the risk of publication bias evaluated through funnel plot asymmetry and Egger tests. We acknowledge that many other factors including heterogeneity, differences in methodological quality, and selective reporting may produce funnel plot asymmetry (77).

The influence of each study on a meta-analysis estimate was investigated through influence analysis, where each individual study is omitted in turn and the meta-analysis re-estimated using a random effects model. For publications that included more than one subgroup of participants with dementia, the largest subgroup was included in the influence analysis.

There was no funding source for this study and the corresponding author had full access to all of the data in the study and had final responsibility for the decision to submit for publication.

## Results

### Systematic Review

Five database searches resulted in 18,456 retrieved abstracts after removal of duplicates (Figure 1). Two hundred thirty of these publications qualified for full-text screening after examination by title and abstract. Forty-eight studies qualified for inclusion in the final qualitative analysis and 19 of these publications qualified for inclusion in one or multiple meta-analyses (Dementia: *n* = 617, Control: *n* = 406) (**Table 7**).

Nineteen studies (39%) enrolled participants only with ad-related diagnoses. Table 2 describes the technical specifications of devices used in individual studies. Thirty-four studies (70%) tested participants using a wrist-worn actigraph. The average assigned duration of wear was 8.26 days (range: 6 min−28 days). Forty (83%) studies used accelerometry as the main measurement of activity. One study used an accelerometer with a gyroscope, while one further study used an accelerometer, gyroscope and magnetometer. Six studies (12%) used activity monitors which did not state the type of measurement modality.

**Table 2:** Technical specifications of wearable devices used in individual studies.

**Source**	**Measurement method [Author reported]**	**Product/company name**	**• Device placement/** **• Length of monitoring**
Aharon-Peretz et al. (7)	Actigraph	Ambulatory Monitoring Inc., Ardsley, NY	• Wrist • 8 days
Ahmed et al. (10)	Tri-axial accelerometer	Actiheart device–CamNtech	• Chest/left mid-clavicular line • 7 days
Anderson et al. (14)	Actigraph	Actiwatch; Cambridge Neurotechnology, Cambridge, UK	• Wrist • 28 days
Brown et al. (16)	Actigraph	ActiGraph ActiSleep monitor (ActiGraph LLC, 2013)	• Non-dominant wrist • 72 h
Carvalho-Bos et al. (17)	Actigraph	Actiwatch; Cambridge Neurotechnology, Cambridge, UK	• Non-dominant wrist • 2 consecutive weeks
David et al. (19)	Actigraph	Actiwatch-L/MiniMitter	• Non-dominant wrist • 75 consecutive minutes
David et al. (20)	Actigraph	Micro- Mini MotionLogger, Ambulatory-Monitoring, Inc., Ardsley, NY	• Non-dominant wrist • Seven consecutive 24-hour periods
Eggermont and Scherder (21)	Actigraph	Actigraph activity monitor; Cambridge Neurotechnology Ltd. Cambridge, England	• Wrist • 4 consecutive days
Fetveit and Bjorvatn (22)	Actigraph	Actiwatch portable recorder (Cambridge Neurotechnology Ltd, UK)	• Dominant hand (Due to paralysis in the dominant arm, two residents wore the actigraph on the non-dominant arm) • 14 Days
Fleiner et al. (24)	Triaxial accelerometer, gyroscope and magnetometer	uSense sensors (FARSEEING EU-Consortium, 2015)	• Lower back • 72 consecutive hours
Gehrman et al. (26)	Actigraph/photometric transducer	Actillume Monitor (Ambulatory Monitoring, Ardsley, NY)	• Dominant wrist • 3 Days
Ghali et al. (27)	Electronic motion detection monitor	Not stated	• Above left elbow • 48 h
Harper et al. (28)	Activity monitor	AM-16, Ambulatory Monitoring, Inc. Ardsley, NY	• Ankle • 72 h
Harper et al. (29)	Activity monitor	AM-16, Ambulatory Monitoring, Inc. Ardsley, NY	• Nondominant ankle • 72 h
Hatfield et al. (30)	Actigraph	Actiwatch; Cambridge Neurotechnology, Cambridge, UK	• Wrist • 28 days
Hooghiemstra et al. (31)	Actigraph	The Actiwatch-4 (AW4) activity monitor (Cambridge Neurotechnology Ltd, Cambridge, UK)	• Dominant wrist • 7 consecutive days
Ijmker and Lamoth, (35)	Tri-axial accelerometer	DynaPort MiniMod	• Lower Back • Two 3-min periods
Iwata et al. (36)	Tri-axial accelerometer	HJA-350IT; Omron, Kyoto, Japan	• 2–3 months during waking hours
James et al. (37)	Actigraph	Actical, Mini Mitter	• Non-dominant wrist • 2–16 days
Kodama et al. (38)	Activity monitor	The Actiwatch-2 (AW2) Philips Respironics Inc.	• Non-dominant wrist • 7 days
König et al. (39)	Actigraph	Prototype, Philips Research Laboratories Europe	• Wrist • Duration of walking tasks
Kuhlmei et al. (41)	Actigraph	Actiwatch Mini, Cambridge Neurotechnology	• Wrist • 5 days
La Morgia et al. (43)	Actigraph	Actigraph Mini Motionlogger, Ambulatory Monitoring, Inc. Ardsley, NY	• Non-dominant wrist • 7 days
Lamoth et al. (44)	Tri-axial accelerometer	DynaPort MiniMod	• Lower back • Duration of testing
Landolt et al. (45)	Actigraph	Actiwatch, Cambridge Technology	• Wrist • 2 weeks
Lee et al. (46)	activity monitor	Mini-Logger (Mini-Mitter company)	• Wrist • 96 h
Leger et al. (47)	Actigraph	Motionwatch 8 (MW8, Camntech, Cambridge, UK)	• Non-dominant wrist • 14 days
McCurry et al. (49)	Actigraph	Actillume wrist-movement recorder (Ambulatory Monitoring, Inc., Ardsley, NY)	• Wrist • 7 days
Merrilees et al. (50)	Actigraph	MiniMitter Actiwatch monitors (AW-64)	• Non-dominant wrist • 2 weeks
Most et al. (52)	Actigraph	Actiwatch; Cambridge Neurotechnology, Cambridge, UK	• Non-dominant wrist • 2 weeks
Moyle et al. (53)	Tri-axial accelerometer	SenseWear® Professional 8.0 activity armband (Temple Healthcare, BodyMedia, Inc)	• Upper non-dominant arm • Monday to Saturday
Mulin et al. (54)	Actigraph	Micro- Mini MotionLogger, Ambulatory-Monitoring, Inc., Ardsley, NY	• Non-dominant wrist • 7 days
Murphy et al. (55)	Tri-axial accelerometer	Sensewear Armband, Body Media	• Upper left arm • 7 days
Olsen et al. (56)	Actigraph	ActiSleep+, Actigraph, Pensacola, USA	• Left wrist • 7 days
Paavilainen et al. (57)	Telemonitoring and actigraphy system	Information Security Technology (IST) Vivago system	• Wrist • 9–113 days
Pollak and Stokes, (58)	Activity Monitor	MiniMotionlogger recorder (Ambulatory Monitoring, Inc.)	• Non-dominant wrist • 9 days
Rindlisbacher and Hopkins, (60)	Ambulatory monitoring device	Not stated	• Above left elbow (in shirt) • Four consecutive days
Schwenk et al. (61)	Accelerometer/gyroscope	Physilog (BioAGM, CH)	• Chest • 24 h
Valembois et al. (62)	Actigraph	Vivago, Vivago Oy, Espoo, Finland	• Non-dominant wrist • 10 days
Van Alphen et al. (63)	Tri-axial accelerometer	The Actiwatch-4 (AW4) activity monitor (Cambridge Neurotechnology Ltd, Cambridge, UK)	• Dominant wrist • 6 days
van Someren et al. (64)	Actigraph	Not stated	• Wrist • 155 h
Varma and Watts, (65)	Actigraph	Actigraph GT3X+	• Dominant hip • 7 days
Viegas et al. (66)	Actigraph	Basic Mini-Motionlogger Actigraph, Ambulatory Monitoring, Inc.	• Wrist • Five 24-h periods
Volicer et al. (67)	Activity monitor	AM-16 Ambulatory Monitoring, Ardsley NY	• Waist • 72 h
Wams et al. (68)	Actigraph	Actiwatch 7, CamNTech Ltd.	• Non-dominant wrist • 3 weeks
Weissova et al. (69)	Actigraph	Actiwatch, AW4 model, Cambridge Neurotechnology Ltd.	• Non-dominant wrist • 21 days
Wirz-Justice et al. (70)	Actigraph with luxmeter	Actiwatch-L, Cambridge Neurotechnologies	• Non-dominant wrist • 10–26 days
Yesavage et al. (71)	Actigraph	Ambulatory Monitoring Systems, Inc. Ardsley, NY	• Non-dominant wrist • 6 days

### Daily Activity

**Figure 2:**
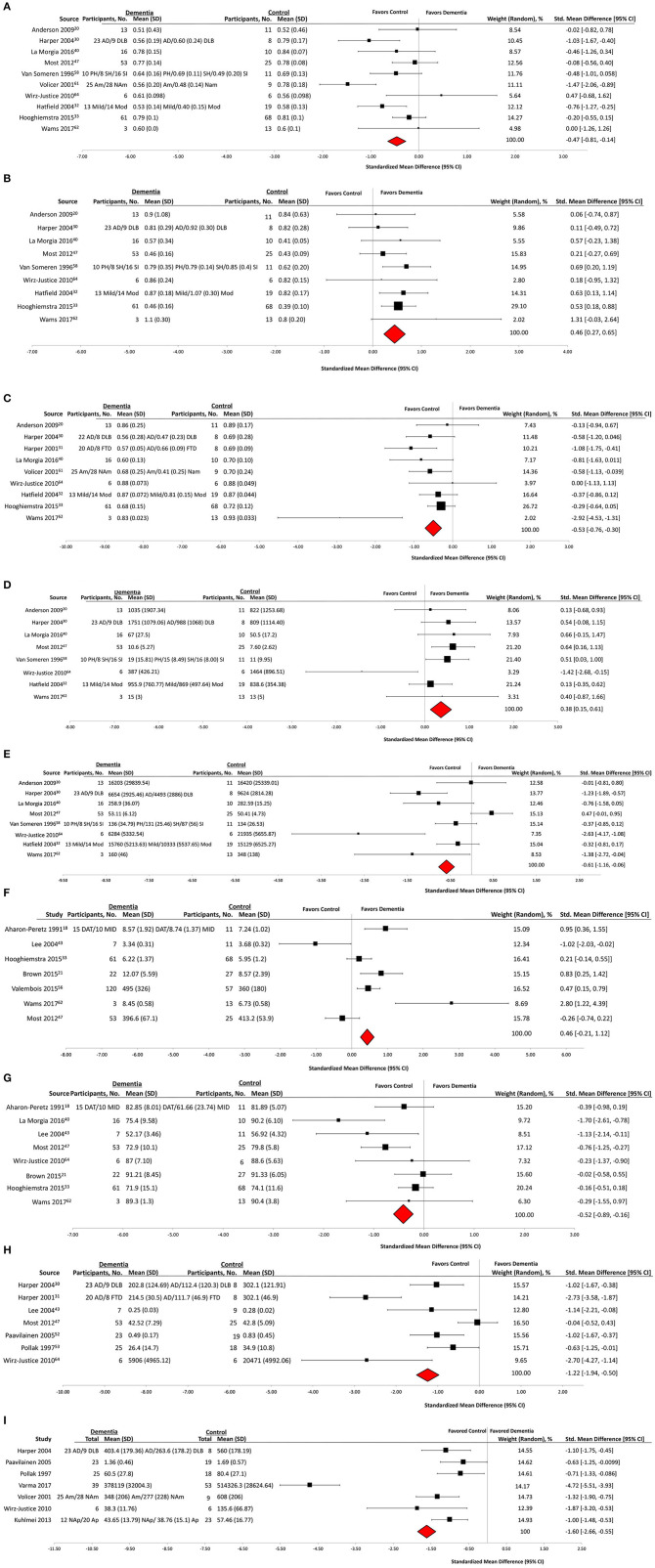
Actigraphy outcomes in observational case control studies of wearable technology. **(A)** Interdaily stability, **(B)** interdaily variability, **(C)** relative amplitude, **(D)** activity of least active 5 h, **(E)** Activity of most active 10 h, **(F)** total sleep time, **(G)** sleep efficiency, **(H)** amplitude, and **(I)** daytime activity.

Of the 48 included studies, 23 (47%) groups reported outcome data on daily activity counts as measured by actigraphy. Qualitative analysis showed that activity counts were presented in a number of different ways (Table 3). Significant associations of activity counts with other measures, or differences in activity between individuals with dementia and control groups, were reported for the measures of daily activity (eight groups, 34%), peak daily activity (two groups, 8%), mean activity counts (five groups, 21%), daytime activity (five groups, 21%), night time activity (one group, 4%), number of immobile hours (one group, 4%), and activity patterns (three groups, 13%). Quantitative analysis demonstrated that participants with dementia had a significantly lower mean daytime activity counts compared to controls (mean difference, −1.60; 95% CI, −2.66 to −0.55) (Dementia: *n* = 210, Control: *n* = 136) (Figure 2I).

**Table 3:** Specific outcome measures of daily activity reported by included studies.

**Source**	**Daily activity**	**Peak daily activity**	**Mean activity counts**	**Daytime activity**	**Nighttime activity**	**Immobile hours**	**Activity patterns**
Aharon-Peretz et al. (7)	+						
Ahmed et al. (10)			+				
Carvalho-Bos et al. (17)				+			
David et al. (19)			+				
David et al. (20)				+	-		
Eggermont and Scherder (21)				+	-		
Ghali et al. (27)		+					
Harper et al. (28)	+				–		
Harper et al. (29)	+				+		
James et al. (37)	+						
Kuhlmei et al. (41)				+			
Merrilees et al. (50)						+	
Moyle et al. (53)	+						
Mulin et al. (54)			+				
Olsen et al. (53)							+
Paavilainen et al. (57)			+				
Pollak and Stokes. (58)				+	–		
Rindlisbacher and Hopkins (60)							+
Wirz-Justice et al. (70)			+				
Valembois et al. (62)	+						
van Alphen et al. (63)							+
Volicer et al. (67)	+						
Varma and Watts. (65)	+	+					

*+ indicates a significant association or difference reported*.

*–indicates no significant association or difference reported*.

### Wearable Actigraphy for Sleep Derived Measures

Of the 48 included studies, 31 (64%) groups reported outcome data on sleep characteristics as measured by actigraphy (Table 4). Wake after sleep onset (WASO) was reported by 11 groups: six (55%) reported a significant association or difference in dementia subjects. Total sleep time (TST) was reported by 16 groups: nine (56%) reported a significant association or difference in dementia subjects. Sleep efficiency (SE) was reported by 12 groups: five (42%) reported a significant association or difference in dementia subjects. Participants with dementia had statistically significant lower mean sleep efficiency than controls (mean difference, −0.52; 95% CI, −0.89 to −0.16) (Dementia: *n* = 193, Control: *n* = 171) (Figure 2G), and no significant difference in mean total sleep time (mean difference, 0.46; 95% CI, −0.21 to 1.12) (Dementia: *n* = 291, Control: *n* = 212) (Figure 2F).

**Table 4:** Specific outcome measures of sleep and circadian rhythm reported by included studies.

**Source**	**WASO**	**TST**	**SE**	**IV**	**IS**	**RA**	**M10**	**L5**	**Mesor**	**Acrophase**	**Amplitude**
Aharon-Peretz et al. (7)		–	+								
Anderson et al. (14)				–	–	–	–	–			
Brown et al. (16)		+	–								
Carvalho-Bos et al. (17)				+	+	+	+	+			
Eggermont and Scherder (21)				+	+	+					
Fetveit and Bjorvatn (22)	+	+	–						–	–	–
Gehrman et al. (26)									–	–	–
Harper et al. (28)				–	+	–	+	+	+	+	+
Harper et al. (29)				+	+	–	+	+	+		+
Hatfield et al. (30)				+	+	+	+	–			
Hooghiemstra et al. (31)	+	+	–	+	–	+					
Kodama et al. (38)				+	+	+					
La Morgia et al. (43)			+	–	–	+	+	–			
Landolt et al. (45)		+									
Lee et al. (46)	–	**–**	–							–	–
Leger et al. (47)	–	+	+	–	–						
McCurry et al. (49)		–									
Most et al. (52)	+	–	+	+	+	–	–	–			
Mulin et al. (50)	+	**–**									
Murphy et al. (55)		+									
Olsen et al. (56)	–	–									
Paavilainen et al. (57)										–	+
Pollak and Stokes (58)										–	+
van Someren et al. (64)				+	+	+	+				
Viegas et al. (66)	+	+									
Volicer et al. (67)					+					+	–
Wams et al. (68)	–	+	–								
Weissova et al. (69)	–	+	–								
Wirz-Justice et al. (70)	–		–	–	–	–	+	+			+
Yesavage et al. (71)	+	–	+						–	–	–

*+indicates a significant association or difference reported*.

*–indicates no significant association or difference reported*.

*IS, Interdaily stability; IV, Intradaily variability; L5, Activity of least active 5 h; M10, Activity of most active 10 h; RA, Relative amplitude; SE, Sleep efficiency; TST, Total sleep time; WASO, Wake after sleep onset*.

### Non-parametric Measurements of Circadian Rhythm Using Wearable Devices

Sixteen (33%) of 48 studies reported non-parametric measurements of circadian rhythm (Table 4). Qualitative analysis revealed that intradaily variability (IV) was reported by 13 groups: eight (61%) reported an association or difference in dementia groups. Interdaily stability (IS) was reported by 14 groups: nine (64%) reported an association or difference in dementia subjects. Relative amplitude (RA) was reported by 12 groups: seven (58%) reported an association or difference in dementia subjects. Activity of most active 10 h (M10) was reported by nine groups: seven (77%) reported an association or difference in dementia subjects. Activity of least active 5 h (L5) was reported by eight groups: four (50%) reported an association or difference in dementia groups.

Participants with dementia had significantly lower mean values than controls on IS (mean difference, −0.47; 95% CI, −0.81 to −0·14) (Dementia: *n* = 298, Control: *n* = 180) (Figure 2A), RA (mean difference, −0.53; 95% CI, −0.76 to −0.30) (Dementia: *n* = 237, Control: *n* = 152) (Figure 2C), and M10 (mean difference, −0.61; 95% CI, −1.16 to −0.06) (Dementia: *n* = 184, Control: *n* = 103) (Figure 2E) outcomes. Participants with dementia had statistically significantly higher mean values than controls on IV (mean difference, 0.46; 95% CI, 0.27–0.65) (Dementia: *n* = 245, Control: *n* = 171) (Figure 2B) and L5 (mean difference, 0.38; 95% CI, 0.15–0.61) (Dementia: *n* = 184, Control: *n* = 103) (Figure 2D) outcomes.

### Cosinor Analysis of Circadian Rhythm Using Wearable Devices

Nine (19%) out of the 48 groups reported a cosinor analysis of circadian rhythm. Qualitative analysis (Table 4) showed that midline estimating statistic of rhythm (mesor) was reported by five groups: two (40%) reported a significant association or difference in dementia subjects. Amplitude of the cosinor wave was reported by 10 groups: five (50%) reported an association or difference in dementia subjects. Acrophase was reported by eight groups: two (25%) reported an association or difference in dementia subjects. Quantitative analysis was only performed on the amplitude of the cosinor wave. It revealed that subjects with dementia had a significantly lower mean amplitude than controls (mean difference, −1.22; 95% CI, −1.94 to −0.50) (Dementia: *n* = 174, Control: *n* = 93) (Figure 2H).

### Wearable Actigraphy for Gait Derived Measures

Of the 48 included studies six (12%) groups reported outcome data on actigraphy to measure posture and gait characteristics (Table 5). Qualitative analysis showed that all six (100%) reported an association or difference in dementia subjects. These studies each reported a different measure of gait or walking activity, and thus a meta-analysis was not possible.

**Table 5:** Specific outcome measures of gait and walking activity reported by included studies.

**Source**	**Gait**	**Gait speed**	**Walking speed**	**Cadence**	**Step variance**	**Dual tasking**	**Walking duration**	**Physical activity**
Aharon-Peretz et al. (7)	+							
Harper et al. (29)		+						
Iwata et al. (36)			+	–	–			
La Morgia et al. (43)						+		
Van Alphen et al. (63)							+	
Volicer et al. (67)								+

*+, Indicates a significant association or difference reported*.

*–, Indicates no significant association or difference reported*.

### Risk of Bias Within Studies

Average rating of methodological quality of included studies was 15·54 points (*SD* = 1·47). The median and mode were both 16 points, with a range of 12–18 (Table 6).

**Table 6:** Characteristics and major findings of included studies.

**Source**	**Study design**	**Participants (*n*)**	**Age, mean (SD or range), y**	**Major findings**	**Quality score^†^**
Aharon-Peretz et al. (7)^‡^	Prospective Case control	MID (10) AD (15) Control (11)	MID 75.9 (8.2) AD 72.8 (6.3) Control 69.0 (3.4)	Groups with dementia demonstrated significant differences in sleep efficiency and total daily activity but not total sleep time.	13
Ahmed et al. (10)	Prospective case control	FTD (19) AD (13) Control (16)	Not Stated	Decreased activity levels observed in dementia groups compared to controls. Increased stressed and resting heart rates in dementia groups compared to controls.	17
Anderson et al. (14)^‡^	Prospective case control	FTD (13) Control (11)	FTD 63.9 (8.8) Control 66.8 (5.7)	Increase in nocturnal activity and decrease in morning activity in dementia group compared to controls. No significant overall difference in non-parametric analysis of circadian rhythm between dementia group and controls.	18
Brown et al. (16)^‡^	Prospective cross sectional	DEM AC (22) Control (27)	Not Stated	Less robust sleep wake rhythms, increased total sleep time, and increased time spent in bed in group with dementia but no difference in sleep efficiency as compared to participants without dementia.	17
Carvalho-Bos et al. (17)	Prospective cohort	AD (57) VaD (13) DEM AC (10)	85.5 (5.9)	A lower level of cognitive functioning as measured by the MMSE and higher functional impairment were associated with a less stable rest-activity rhythm.	17
David et al. (19)	Prospective Case control	AD (32) Control (15)	AD 78.6 (7.4) Control 73.1 (6.0)	Lower activity levels in dementia group compared to controls.	13
David et al. (20)	Prospective cohort	AD (107)	AD 77.2 (6.7)	Participants with dementia and apathy had lower daytime activity levels than those without apathy.	16
Eggermont and Scherder (21)	Prospective cohort	DEM AC (76)	DEM AC 84.9	No association between cognition and motor activity.	17
Fetveit and Bjorvatn (22)	Prospective cross sectional	DEM AC (23)	DEM AC 86.1 (7.0)	Consistent association between decreased cognition as measured by the MMSE and reduced activity level as well as fragmented sleep.	18
Fleiner et al. (24)	Prospective cross sectional	DEM AC (45)	DEM AC 79 (7)	Low activity levels observed with a wide range of activity patterns in groups with dementia.	16
Gehrman et al. (26)	Retrospective Cross sectional	DEM AC (150)	DEM AC 84.1 (7.8)	No association between rest activity rhythm and severity of dementia as measured by the MMSE, but changes in circadian rhythm observed in those with dementia.	16
Ghali et al. (27)	Prospective cohort	AD (18)	AD 78.8 (6.4)	Time of nocturnal activity peak levels associated with duration of illness (measured in years) in groups with dementia.	16
Harper et al. (28)^‡^	Prospective case control	AD (32) Control (8)	AD 70.2 (1.0) Control 72.8 (2.1)	Increasing AD pathology associated with greater disturbances in circadian activity. Difference in rest-activity between dementia and control groups.	17
Harper et al. (29)^‡^	Prospective case control	DEM AC (38) Control (8)	DEM AC 70.2 (1.0) Control 72.8 (2.1)	Increased nocturnal activity with circadian phase delay observed in participants with AD compared to controls.	15
Hatfield et al. (30)^‡^	Prospective cross sectional	AD (27) Control (19)	AD 68.5 (60–82) Control 71.8 (1.2)	Moderately demented participants show rest activity cycle disturbance when compared to controls. No correlation seen between severity of dementia as measured by the MMSE and rest-activity rhythm.	14
Hooghiemstra et al. (31)^‡^	Prospective cross sectional	DEM AC (61) Control (68)	DEM AC Median 62.5 Control Median 63.0	More rest-activity rhythm fragmentation, more time in bed, more time to transition from wake to sleep in those with early onset dementia than controls.	15
Ijmker and Lamoth (35)	Prospective case control	DEM AC (15) Control (26)	DEM AC 81.7 (6.3) Control 70.6	Changes in gait acceleration in dementia compared to controls	13
Iwata et al. (36)	Prospective case control	DEM AC (14) Control (16)	DEM AC 74.8 Control 73.7	Decreased physical activity in female subjects with dementia as compared to controls	14
James et al. (37)	Retrospective cross-sectional	DEM AC (70) Control (624)	Not stated	Lower levels of total daily activity in subjects with dementia	16
Kodama et al. (38)	Prospective case control	DEM AC (52) Control (66)	DEM AC 78.5 (10.7) Control 72.4 (6.7)	Circadian rhythm parameters significantly differed in subjects with dementia compared to controls	17
König et al. (39)	Prospective case control	AD (23) Control (22)	AD 77 (9) Control 73 (7)	A difference in gait speed under dual task conditions was observed between dementia subjects and controls	17
Kuhlmei et al. (41)^‡^	Retrospective cross-sectional	DEM AC (32) Control (23)	DEM AC 81 Control 78	Reduced daytime activity levels seen in subjects with dementia	12
La Morgia et al. (43)^‡^	Prospective case control	AD (16) Control (10)	AD 70.2 (10.2) Control 65.8 (7.5)	Reduced sleep efficiency seen in subjects with AD as compared to controls	14
Lamoth et al. (44)	Prospective case control	AD (13) Control (13)	AD 82.6 (4.2) Control 79.3 (5.5)	Changes in gait variability in AD compared to controls	16
Landolt et al. (45)	Prospective case control	sCJD (7)	sCJD 65.8 (3.8)	High frequency of sleep wake changes seen in those with sCJD.	15
Lee et al. (46)^‡^	Prospective case control	AD (7) Control (11)	AD 77.0 (4.3) Control 74.2 (5.2)	Mean phase difference (MESOR) was different between those with AD and controls. No significant change was seen in mean acrophase or mean amplitude of temperature.	15
Leger et al. (47)	Retrospective cross sectional	AD (208)	AD 73 (11.6)	Increased time spent in bed in those with moderate AD as measured by the MMSE compared to those with mild AD.	16
McCurry et al. (49)	Prospective cohort	AD (44)	AD 78.8 (7.2)	Significant variation seen in all sleep measures both between and within all subjects	14
Merrilees et al. (50)	Prospective cohort	FTD (22)	FTD 63.8	In patients with FTD, apathy was associated with lower activity levels and greater number of bouts of immobility	15
Most et al. (52)^‡^	Prospective case control	AD (55) Control (26)	AD 70.4 (3.2) Control 73.0 (4.4)	Longer sleep onset latency and decreased sleep efficiency was seen in subjects with AD compared to controls.	15
Moyle et al. (53)	Retrospective cross sectional	DEM AC (192)	DEM AC 85.5 (7.7)	No significant correlation seen between level of cognitive impairment as measured by the MMSE and activity and sleep patterns over 24 h.	16
Mulin et al. (54)	Prospective cohort	AD (103)	AD 76.9 (7.2)	Subjects with apathy demonstrated more time spent in bed during the night, and lower daytime motor activity than those without apathy.	14
Murphy et al. (55)	Retrospective cross-sectional	DEM AC (20)	DEM AC 78.7 (1.8)	Energy expenditure inversely related to time spent lying down and sleep duration.	15
Olsen et al. (56)	Retrospective cross-sectional	DEM AC (193)	DEM AC 83.6	Decreased activity in nursing home subjects with dementia compared to home dwelling subjects with dementia.	16
Paavilainen et al. (57)^‡^	Prospective case control	DEM AC (23) Control (19)	DEM AC 84.3 (9.5) Control 81.5 (9.0)	Subject with dementia demonstrated lower daytime and higher nocturnal activity than controls	16
Pollak and Stokes (58)^‡^	Prospective case control	DEM AC (25) Control (18)	DEM AC 80.7 (7.9) Control 73.7 (7.2)	Less activity and flat cosine analysis of circadian rhythm in groups with dementia when compared to controls	18
Rindlisbacher and Hopkins (60)	Prospective cohort	AD (12)	AD 79.4	Variability in 24-h peaks of activity correlated with years of illness	16
Schwenk et al. (61)	Prospective cohort	DEM AC (77)	DEM AC 81.8 (6.3)	Actigraph derived “walking bouts average duration” demonstrated a positive predictive value for future falls in subjects with dementia	17
Valembois et al. (62)^‡^	Prospective cross sectional	DEM AC (126) Control (57)	All Participants 84.9 (6.8)	Decreased motor activity in subjects with dementia who demonstrate apathy and anxiety. No association between agitation and motor activity.	15
Van Alphen et al. (63)	Retrospective cross-sectional	DEM AC (146)	DEM AC 83.0 (7.6)	Increased sedentary levels and decreased physical activity levels in subjects with dementia who were institutional dwelling	17
van Someren et al. (64)^‡^	Prospective case control	DEM AC (34) Control (11)	DEM AC 74.7 Control 72 (1.2)	Less stable rest-activity rhythm in institutionalized subjects with dementia compared to subjects cared for at home and controls	15
Varma and Watts (65)^‡^	Prospective case control	AD (39) Control (53)	AD 73.5 (7.9) Control 73.2 (6.5)	Decreased physical activity and changes in activity patterns seen in dementia subjects compared to controls	15
Viegas et al. (66)	Retrospective cross sectional	DEM AC (104)	DEM AC 82.9 (8.4)	Average of 476min sleep per 24 h in subjects with dementia.	18
Volicer et al. (67)^‡^	Prospective case control	AD (25) Control (9)	AD 71.0 (60–88) Control 73.4 (67–83)	A high percentage of nocturnal activity and less diurnal motor activity in subjects with AD compared to controls	17
Wams et al. (68)^‡^	Retrospective cross sectional	AD (29) Control (14)	AD 77.7 (7.6) Control 73.8 (4.6)	AD patients demonstrated longer time in bed, longer sleep duration, and lower amplitude than controls. No difference between groups in sleep quality	17
Weissová et al. (69)	Prospective case control	AD (4) Control (4)	Not stated	No difference in sleep parameters in participants with AD compared to controls	16
Wirz-Justice et al. (70)^‡^	Prospective cross sectional	KP (6) Control (6)	KP 66.8 Control Not Stated	Longer nocturnal rest duration and lower daytime activity level in participants with KP compared to controls	14
Yesavage et al. (71)	Retrospective cross sectional	AD (61)	AD 71.4 (8.1)	AD participants show worsening in parameters of nocturnal sleep but no change in rest/activity circadian rhythm over time	13

*AD, Alzheimer's Disease; DEM AC, Dementia All Cause; FTD, Frontotemporal Dementia; KP, Korsakoff Psychosis; MID, Multi-Infarct Dementia; MMSE, Mini-Mental State Examination; sCJD, sporadic Creutzfeldt-Jakob Disease; SD, Standard Deviation; VaD, Vascular Dementia; y, Years*.

† *Refers to endpoints reported by authors*.

‡ *Study included in one or multiple meta-analyses*.

### Meta-Analysis and Heterogeneity

Low between study heterogeneity (*I*^2^ < 50%) was observed for analyses of IV, RA, and L5 variables (Table 7). Moderate to high between study heterogeneity (*I*^2^ > 50%) was observed for analyses of IS, TST, amplitude, M10, SE, and daytime activity. Meta-regression or subgroup analyses were performed for all actigraphy measures with a moderate to high heterogeneity (*I*^2^ > 50%) which included IS, TST, Amplitude, M10, SE, and daytime activity. Type of dementia, mean age, study design and quality score were all investigated as explanatory variables. Subgroup analyses indicate that effect estimates vary markedly between dementia subtypes for variables M10 and SE, suggesting differences in dementia type between studies may account for some of the heterogeneity observed in meta-analyses of M10 and SE measurements.

**Table 7:** Combined effect estimates and heterogeneity for actigraphy outcomes between dementia and control samples.

**Actigraphy measure**	**Included studies, no**.	**Dementia subjects, no**.	**Healthy subjects, no**.	**Pooled mean difference, random-effects model (95% CI)**	***Q***	***I*^**2**^ (95% CI)**
IS	10	298	180	−0.47 (−0.81, −0.14)^†^	24.86 (9 *df*)^§^	64 (29, 82)
IV	9	245	171	0.46 (0.27, 0.65)^†^	6.61 (8 *df*)	0 (0, 65)
RA	9	237	152	−0.53 (−0.76, −0.30)^†^	15.39 (8 *df*)	48 (0, 76)
L5	8	184	103	0.38 (0.15, 0.61)^§^	11.21 (7 *df*)	38 (0, 72)
M10	8	184	103	−0.61 (−1.16, −0.06)^§^	30.32 (7 *df*)^†^	77 (54, 88)
TST	7	291	212	0.46 (−0.21, 1.12)	30.31 (6 *df*)^†^	80 (60, 90)
SE	8	193	171	−0.52 (−0.89, −0.16)^§^	15.44 (7 *df*)^§^	55 (0, 80)
Amplitude	7	174	93	−1.22 (−1.94, −0.50)^‡^	36.34 (6 *df*)^†^	83 (67, 92)
Daytime Activity	7	210	136	−1.60 (−2.66, −0.55)^§^	81.82 (6 *df*)^†^	93 (87, 96)

*df, degrees of freedom; IS, interdaily stability; IV, intradaily variability; I^2^, percentage of variation across studies due to heterogeneity; L5, activity of least active 5 h; M10, activity of most active 10 h; No., number; Q, Cochran's Q weighted sum of squares of effect size estimates subtracted by their mean; RA, relative amplitude; SE, sleep efficiency; TST, total sleep time*.

† *p < 0.0001*.

‡ *p < 0.001*.

§ *p < 0.05*.

### Risk of Publication Bias Across Studies for Meta-Analysis

**Figure 3:**
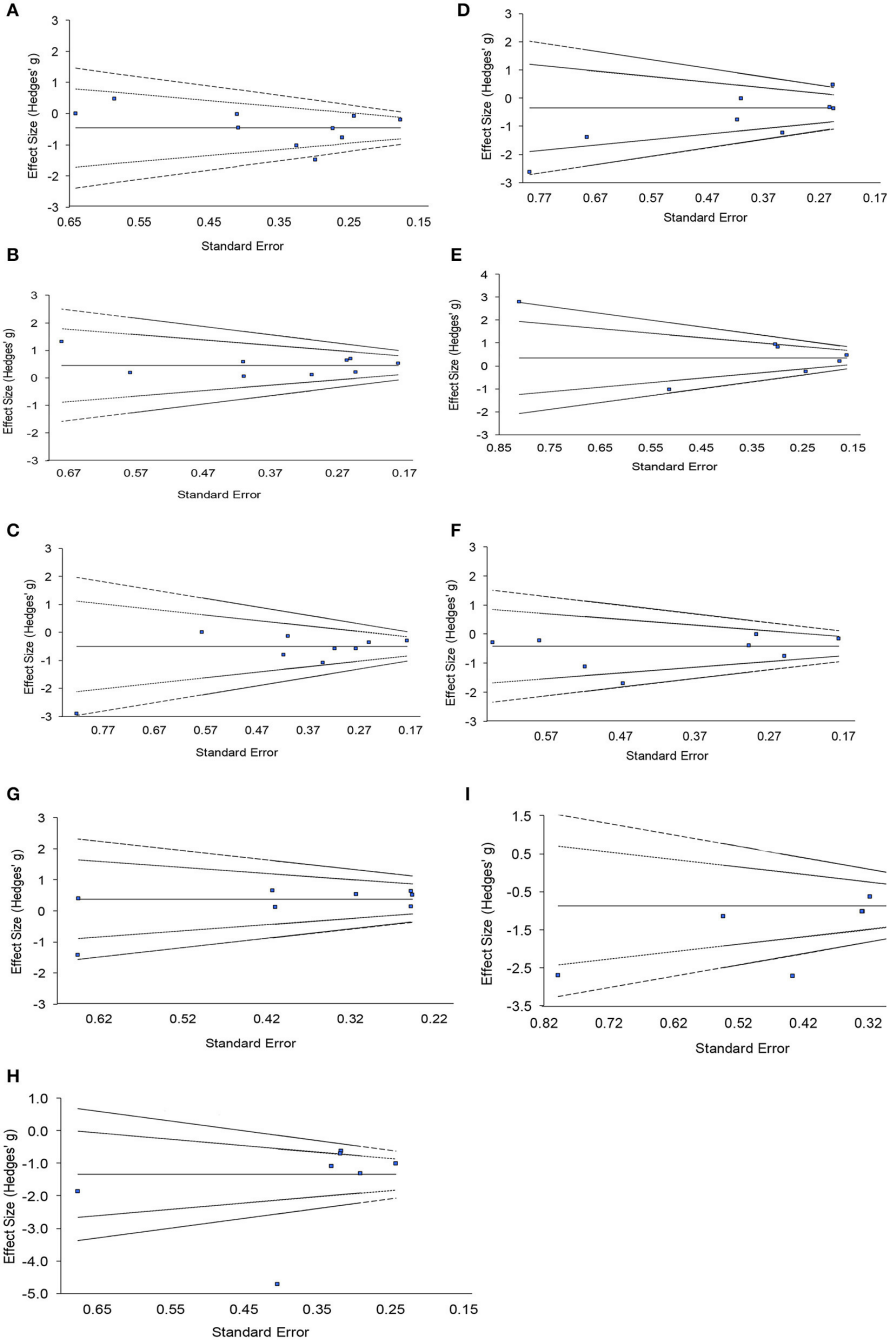
Funnel plots with pseudo 95 and 99.7% confidence intervals assessing publication bias of included studies for nine actigraphy measures. **(A)** Interdaily stability, **(B)** intradaily variability, **(C)** relative amplitude, **(D)** activity of most active 10h, **(E)** total sleep time, **(F)** sleep efficiency, **(G)** activity of least active 5h, **(H)** daytime activity, **(I)** amplitude.

Funnel plots for each variable investigated using random effects meta-analysis are provided in Figure 3. These plots were constructed with a measure of study size on the x-axis and a measure of effect size on the y-axis. Dashed lines represent the pseudo 95 and 99.7% confidence limits about the effect estimate (solid line). Funnel plot asymmetry was observed for all but two variables (IV and RA), and significant Egger tests observed for M10 (*p* = 0.0057) and amplitude variables (*p* = 0.0078), suggesting evidence of publication bias for these measurements.

### Investigation of Influential Studies

**Figure 4:**
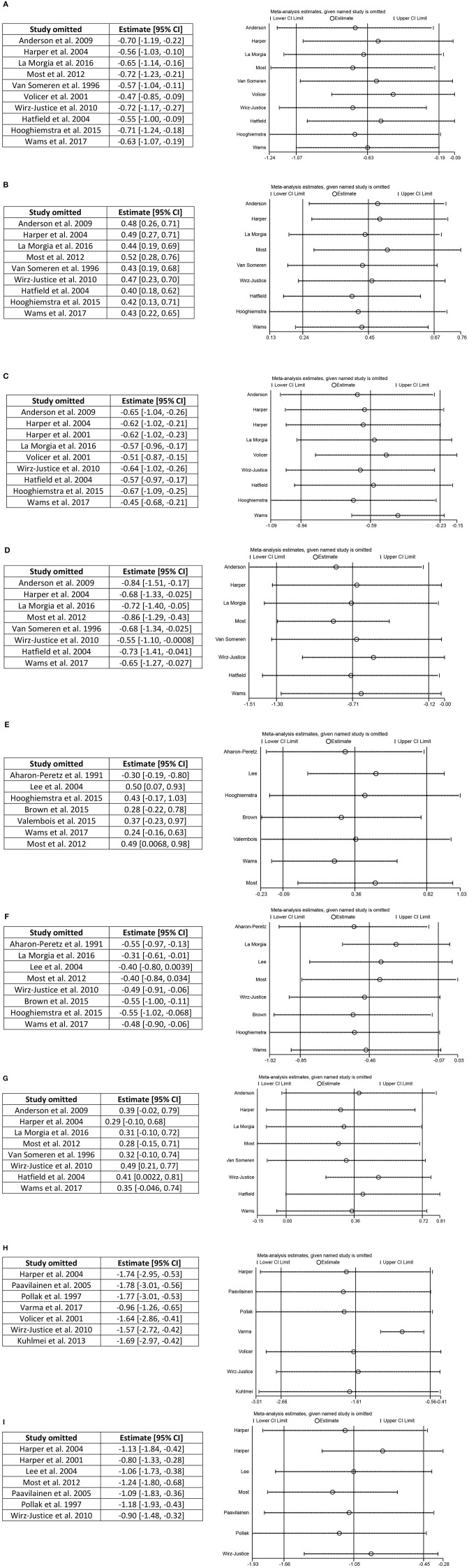
Influence analysis. **(A)** Interdaily stability, **(B)** intradaily variability, **(C)** relative amplitude, **(D)** activity of most active 10 h, **(E)** total sleep time, **(F)** sleep efficiency, **(G)** activity of least active 5 h, **(H)** daytime activity, and **(I)** amplitude.

The impact of each study on a meta-analysis estimate was investigated through influence analysis. Influence analysis shows that meta-analysis estimates are generally robust (Figure 4), excluding meta-analysis of daytime activity, where the pooled estimate decreases in magnitude markedly and precision of the estimate improves with exclusion of Varma and Watts (65). Even with exclusion of this influential study, the pooled estimate remains significant and shows the same direction of effect as in the full meta-analysis.

## Discussion

From our systematic review of the literature we found 48 articles which met our inclusion criteria of wearable technology use in patients with dementia for the measurement of physiological parameters. Wearable devices were utilized most extensively to measure circadian rhythm, measurement of the sleep wake cycle and daily activity. In the studies which were analyzed using forest plots, groups of participants with dementia were less active then controls, had a difference in their sleep wake cycle and showed differences in their circadian rhythms when compared to control groups. To our knowledge, this study is the first systematic review and meta-analysis of wearable device testing in participants with dementia.

### Wearable Devices to Measure Sleep and Circadian Rhythm

The use of actigraphy to measure sleep was the most commonly reported outcome. Participants with dementia demonstrated reduced sleep efficiency as compared to controls. There was also a significant difference between individuals with dementia and controls on non-parametric measures of circadian rhythm including IV, IS, and RA, however it should be noted that for some measures the combined effects were substantially weighted by the results of Hooghiemstra et al. (31). Meta-analysis of the amplitude measure of circadian rhythm cosinor analysis also demonstrated a moderate but statistically significant difference between groups. Again, a high level of heterogeneity between studies was observed for this outcome measure. Despite evidence of the utility of wearable actigraphy in sleep monitoring, consistent outcome measures and methods of analyzing sleep data and circadian rhythm have not been universally agreed upon (2). In order for actigraphy to become routinely used in clinical and drug treatment trials, consistent outcome measures are needed and, as shown in this meta-analysis, may provide a useful endpoint for patients with dementia.

### Wearable Devices and Daily Activity

When using wearable devices to measure daily activity, those with dementia had significantly lower daily activity counts than controls. This effect was demonstrated despite across-study variation in methods of calculating daytime activity including peak activity counts, mean activity, and daily activity. A meta-analysis of studies measuring daily activity showed that subjects with dementia demonstrate significantly less daily activity as compared to controls. Four groups reported no differences in nocturnal activity between subjects with dementia and controls. It should be noted that two of these studies did not recruit a control group, but instead compared participants with dementia to their caregivers [McCurry et al. (49) and Merrilees et al. (50)]. Physical activity has been examined in longitudinal studies and found to be associated with both development of dementia as well as disease progression (78). There is increasing evidence that physical activity and exercise as part of multi-domain interventions holds benefit for patients with dementia (79). However, as demonstrated in this review, definitions of physical activity differ significantly between studies and daily activity counts measured by wearable devices are not definite indicators of beneficial exercise, but merely of movement. Some researchers have attempted to quantify daily activity counts into variables such as energy expenditure, and this measure was also reduced in participants with dementia as compared to controls (55). With the growing availability of consumer wrist worn devices for movement and activity tracking, the use of daily activity measurements provides a potential novel end point for large scale clinical trials in dementia.

### Wearable Devices and Gait

Analysis of gait behavior was studied by six groups. Significant differences between controls and those with dementia were reported by all groups for multiple aspects of the gait cycle and behavior. However, due to the variation in reported outcomes, a quantitative analysis could not be performed and conclusions regarding the use of wearable devices for the study of gait could not be reliably made. It is important to note that gait speed and walking speed were reported as significantly different in subjects with dementia when compared to controls, while cadence and step variance were not. Lower gait speed in particular has been shown in numerous longitudinal studies to correlate with increased fall risk in older adults (80). Further work to replicate these findings in subjects with dementia is warranted.

### Limitations

The main limitation of the meta-analysis was the between-study heterogeneity (Table 7). Given differences in characteristics of study design such as duration of testing, wearable device type, and diagnosis, statistical heterogeneity was expected between publications included in each meta-analysis. Despite this, effect size comparisons between healthy volunteers and participants with dementia were generally consistent in direction between studies. Methodological considerations specifically for actigraphy testing in dementia have been more thoroughly addressed in a clinical review (81). Also, all papers included in this review corresponded to definitions of both all cause dementia and wearable devices which were agreed upon by the author group. As a result, studies which did not conform to these definitions have been excluded and the effect these may have had on the analysis cannot be quantified. Lastly not all devices used have been compared to gold—standard clinical testing and their methods of measurement may differ and therefore their reported differences should be interpreted with caution.

## Conclusions and Implications

In conclusion this systematic review and meta-analysis has shown that the wearable devices studied demonstrate differences in those with dementia when compared to controls. Specifically, it provides evidence that wearable devices demonstrate a utility in measuring levels of activity, changes in circadian rhythm, and changes in the sleep wake cycle. Included studies were limited by their heterogeneity, the lack of classification of dementia sub-type and stage, as well as the lack of confirmatory clinical trials. Further work is warranted to correlate these findings with clinical changes which may represent surrogate digital end-points such as the neuro-psychiatric manifestations associated with circadian rhythm changes and the loss of mobility associated with decreased activity.

## Data Availability Statement

All datasets generated for this study are included in the article/Supplementary Material.

## Author Contributions

JB: concept and design. JB, AC, and RP: acquisition, analysis, or interpretation of data, and drafting of the manuscript. JB, AC, RP, NK, and KT: critical revision of the manuscript for important intellectual content, administrative, technical, or material support. AC: statistical analysis. KT: obtained funding. JB and KT: supervision. AC had full access to all of the data in the study and takes responsibility for the integrity of the data and the accuracy of the data analysis. All authors contributed to the article and approved the submitted version.

## Conflict of Interest

The authors declare that the research was conducted in the absence of any commercial or financial relationships that could be construed as a potential conflict of interest.
