# Corrigendum: Evaluation of Wearable Technology in Dementia: A Systematic Review and Meta-Analysis

**DOI:** 10.3389/fmed.2021.659639

**Published:** 2021-03-11

**Authors:** Alanna C. Cote, Riley J. Phelps, Nina Shaafi Kabiri, Jaspreet S. Bhangu, Kevin “Kip” Thomas

**Affiliations:** ^1^Department of Anatomy and Neurobiology, Boston University Medical Center, Boston, MA, United States; ^2^Department of Genetics and Genomic Sciences, Icahn School of Medicine at Mount Sinai, New York, NY, United States; ^3^Division of Geriatric Medicine, Department of Medicine, Western University, London, ON, Canada

**Keywords:** technology, geriatrics, cognition, sleep, wearable

In the original article, there were mistakes in Tables 1, 3 and 4 as published. Table 1 Column 2 and Table 3 citations were incorrect, and Table 4 was mistakenly included as a duplicate of Table 3. The corrected Tables 1, 3 and 4 appear below.

**Table 1:** Patient setting and diagnostic criteria for 48 observational studies testing wearable technology in participants with dementia.

**Source**	**Diagnostic criteria**	**Setting**
Aharon-Peretz et al. (7)	DSM III-R (63)/NINCDS-ADRDA (64)	Not stated
Ahmed et al. (8)	McKhann et al. (65)/Gorno-Tempini et al. (66)/Rascovsky et al. (67)	Community (home)/In Lab
Anderson et al. (12)	Neary criteria (68)	Community (home)
Brown et al. (14)	Medical record diagnosis	Nursing home
Carvalho-Bos et al. (15)	NINCDS-ADRDA (64)/DSM-IV (69)	Nursing home
David et al. (17)	DSM-IV (69)	Out-patient clinic
David et al. (18)	NINCDS-ADRDA (64)	Out-patient clinic
Eggermont and Scherder (19)	Medical record diagnosis	Nursing home
Fetveit and Bjorvatn (20)	Clinical Dementia Rating (CDR) scale (70)	Nursing home
Fleiner et al. (22)	ICD-10 (71)	Psychiatric hospital
Gehrman et al. (23)	Medical record diagnosis/NINCDS-ARDA (64)	Nursing home
Ghali et al. (24)	DSM-III-R (63)	Dementia treatment Evaluation facility
Harper et al. (25)	NINCDS-ADRDA (64)	Hospital/clinical research center
Harper et al. (26)	NINCDS-ADRDA (64)	Hospital/clinical research center
Hatfield et al. (27)	DSM-IV (69)/ NINCDS-ADRDA (64)	Community (home)
Hooghiemstra et al. (28)	DSM-V (72)/NINCDS-ADRDA (64)/Neary Criteria (68)/McKeith et al. (73)/Pohjasvaara et al. (74)	Not stated
Ijmker and Lamoth (31)	Clinician/ medical record diagnosis	In laboratory
Iwata et al. (32)	DSM-IV (69)/NINCDS-ARDA (64)	Not stated
James et al. (33)	NINCDS-ARDA (64)	Community (home)
Kodama et al. (34)	DSM-III-R (63)	Community (home)
König et al. (35)	International working group−2 criteria (IWG-2) (75)	Memory clinic
Kuhlmei et al. (36)	NINCDS-ADRDA (64)/NINDS-AIREN (76)	Not stated
La Morgia et al. (37)	NINCDS-ADRDA (64)	Not stated
Lamoth et al. (38)	Alzheimer's association criteria	Clinic/hospital
Landolt et al. (39)	Autopsy or biopsy confirmation	Hospital/ nursing home
Lee et al. (40)	NINCDS-ADRDA (64)	Not stated
Leger et al. (41)	DSM-V (72)/ NINCDS-ADRDA (64)/MMSE ≤ 25 and ≥15 (77)/CDR (Score of 0.5, 1, or 2) (70)	Out-patient clinic
McCurry et al. (42)	Family physician/medical record diagnosis	Community (home)
Merrilees et al. (43)	Neary criteria for frontotemporal lobar degeneration (78)	Community (home)
Most et al. (44)	NINCDS- ADRDA (64)	Not stated
Moyle et al. (45)	Medical record diagnosis	Long-term care facility
Mulin et al. (46)	NINCDS- ADRDA (64)	Community (home)
Murphy et al. (47)	MMSE <23 (77)	Nursing home
Olsen et al. (48)	Medical record diagnosis or MMSE <25 (77)	Nursing home/community (home)
Paavilainen et al. (49)	CDR > 0.5 (70)/ MMSE <20 (77)	Nursing home
Pollak and Stokes (50)	Mattis dementia rating scale total score <123 (79) Mattis dementia rating scale memory score <19 (79)	Community (home)
Rindlisbacher and Hopkins (52)	DSM-III-R (63)	Hospital
Schwenk et al. (51)	NINCDS-ADRDA (64)/NINDS-AIREN (76)	Community (home)
Valembois et al. (53)	DSM-IV (69)	Hospital
van Alphen et al. (54)	Medical record diagnosis	Community (home)/nursing home
van Someren et al. (55)	DSM-III-R (63)/NINCDS-ADRDA (64)	Community (home)/nursing home
Varma and Watts (56)	NINCDS-ADRDA (64)	Community (home)
Viegas et al. (57)	DSM-IV (69)/MMSE ≤ 24 (77)	Nursing home
Volicer et al. (58)	NINCDS-ADRDA (64)/DSM-III-R (63)	Hospital
Wams et al. (59)	NINCDS-ADRDA (64)	Community (home)
Weissova et al. (60)	NINCDS-ADRDA (64)	Community (home)
Wirz-Justice et al. (61)	DSM –IV (69)	Hospital
Yesavage et al. (62)	NINCDS-ADRDA (64)	Community (home)

**Table 3:** Specific outcome measures of daily activity reported by included studies.

**Source**	**Daily activity**	**Peak daily activity**	**Mean activity counts**	**Daytime activity**	**Nighttime activity**	**Immobile hours**	**Activity patterns**
Aharon-Peretz et al. (7)	+						
Ahmed et al. (8)			+				
Carvalho-Bos et al. (15)				+			
David et al. (17)			+				
David et al. (18)				+	-		
Eggermont and Scherder (19)				+	-		
Ghali et al. (24)		+					
Harper et al. (25)	+				–		
Harper et al. (26)	+				+		
James et al. (33)	+						
Kuhlmei et al. (36)				+			
Merrilees et al. (43)						+	
Moyle et al. (45)	+						
Mulin et al. (46)			+				
Olsen et al. (48)							+
Paavilainen et al. (49)			+				
Pollak and Stokes. (50)				+	–		
Rindlisbacher and Hopkins (52)							+
Wirz-Justice et al. (61)			+				
Valembois et al. (53)	+						
van Alphen et al. (54)							+
Volicer et al. (58)	+						
Varma and Watts. (56)	+	+					

*+ indicates a significant association or difference reported*.

*–indicates no significant association or difference reported*.

**Table 4:** Specific outcome measures of sleep and circadian rhythm reported by included studies.

**Source**	**WASO**	**TST**	**SE**	**IV**	**IS**	**RA**	**M10**	**L5**	**Mesor**	**Acrophase**	**Amplitude**
Aharon-Peretz et al. (7)		–	+								
Anderson et al. (12)				–	–	–	–	–			
Brown et al. (14)		+	–								
Carvalho-Bos et al. (15)				+	+	+	+	+			
Eggermont and Scherder (19)				+	+	+					
Fetveit and Bjorvatn (20)	+	+	–						–	–	–
Gehrman et al. (23)									–	–	–
Harper et al. (25)				–	+	–	+	+	+	+	+
Harper et al. (26)				+	+	–	+	+	+		+
Hatfield et al. (27)				+	+	+	+	–			
Hooghiemstra et al. (28)	+	+	–	+	–	+					
Kodama et al. (34)				+	+	+					
La Morgia et al. (37)			+	–	–	+	+	–			
Landolt et al. (39)		+									
Lee et al. (40)	–	**–**	–							–	–
Leger et al. (41)	–	+	+	–	–						
McCurry et al. (42)		–									
Most et al. (44)	+	–	+	+	+	–	–	–			
Mulin et al. (46)	+	**–**									
Murphy et al. (47)		+									
Olsen et al. (48)	–	–									
Paavilainen et al. (49)										–	+
Pollak and Stokes (50)										–	+
van Someren et al. (55)				+	+	+	+				
Viegas et al. (57)	+	+									
Volicer et al. (58)					+					+	–
Wams et al. (59)	–	+	–								
Weissova et al. (60)	–	+	–								
Wirz-Justice et al. (61)	–		–	–	–	–	+	+			+
Yesavage et al. (62)	+	–	+						–	–	–

*+indicates a significant association or difference reported*.

*–indicates no significant association or difference reported*.

*IS, Interdaily stability; IV, Intradaily variability; L5, Activity of least active 5 h; M10, Activity of most active 10 h; RA, Relative amplitude; SE, Sleep efficiency; TST, Total sleep time; WASO, Wake after sleep onset*.

The authors apologize for this error and state that this does not change the scientific conclusions of the article in any way. The original article has been updated.
