# Supplementary material for: Evaluation of Wearable Technology in Dementia: A Systematic Review and Meta-Analysis
Source: Front Med (Lausanne). 2021 Jan 11;7:501104. doi: 10.3389/fmed.2020.501104 (PMC7829192; doi:10.3389/fmed.2020.501104)
Supplement: Supplementary file 2 [file Table_2.DOC]

**PICOS Worksheet and Search Strategy**

1. Define your question using **PICOS**: Population, Intervention, Comparison, Outcome, and Study Designs.

**Population**: ___All-cause dementia subjects aged 50+_

**Intervention**: ___ Studies which provided physiological data as measured by wearable technology and did not have an intervention of any kind. ___

**Comparison**: __ Qualitative data such as daytime activity (DA), sleep efficiency (SE), interdaily stability (IS), relative amplitude (RA), activity of most active 10 hours (M10), total sleep time (TST), activity of least active five hours (L5), and intradaily variability (IV).____

**Outcome**: _Adults with dementia were less active, demonstrated increased fragmentation of their sleep-wake cycle and a loss of typical diurnal variation in circadian rhythm as compared to controls as measured by wearable technology__________

**Study Designs**: _Studies had to include participants with a mean age ≥ 50 years and did not include any direct. Studies published before 1970 or translated to English were excluded. Studies that did not provide descriptive statistics for a physiological outcome were excluded. Conference abstracts, review papers, case reports, letters, opinion pieces, editorials, article comments or corrections were excluded. _____

1. Write out your question: __ To systematically review the utility of wearable technology for measurement of physiological parameters in patients with dementia _____

______________________________________________________________________________

1. List the main topics and terms from your question that you can use to search.

________Dementia, Wearable Device, On-body, Actigraphy, Biosensor________________

1. Check any limit that may pertain to your search:

_X_ Age __ Language _X_ Year of publication

1. Type of study/publication you want to include in your search: (From Step 2 of tutorial)

| __ Systematic Review or Meta-Analysis  __ Clinical Practice Guidelines  _X_ Critically Appraised Research Studies | _X_ Individual Research Studies  __ Electronic Textbooks |
| --- | --- |
|  |  |

1. Check the databases you searched:

| _X_ Cochrane Library  __ Joanna Briggs  _X_ EMBASE  _X_ IEEE | __ CINAHL with Full Text  _X_ PubMed Clinical Queries  _X_ PsycInfo |
| --- | --- |

1. What information did you find to help answer your question?

______ Wearable devices were utilized most extensively to measure circadian rhythm, measurement of the sleep wake cycle and daily activity. The use of actigraphy to measure sleep was the most commonly reported outcome. To assess the methodological quality of included studies, we used the checklist provided by Downs and Black. A total quality score is provided for each study. _____________

This form is adapted from: Syrene A. Miller, PICO Worksheet and Search Strategy, National Center for Dental Hygiene Research
